# Clinical efficacy of vestibular assessment and rehabilitation training in peripheral vestibular vertigo

**DOI:** 10.12669/pjms.40.7.8662

**Published:** 2024-08

**Authors:** Huan Wang, Jie Zheng, Fulai Li, Chenfang Feng, Nan Zhang

**Affiliations:** 1 Huan Wang Department of Otolaryngological, Cangzhou People’s Hospital, Cangzhou, 061000, Hebei, China; 2 Jie Zheng Department of Otolaryngological, Cangzhou People’s Hospital, Cangzhou, 061000, Hebei, China; 3 Fulai Li Department of Otolaryngological, Cangzhou People’s Hospital, Cangzhou, 061000, Hebei, China; 4 Chenfang Feng Department of Otolaryngological, Cangzhou People’s Hospital, Cangzhou, 061000, Hebei, China; 5 Nan Zhang Department of Otolaryngological, Cangzhou People’s Hospital, Cangzhou, 061000, Hebei, China

**Keywords:** Vestibular Rehabilitation Training, Peripheral Vestibular Vertigo, Efficacy

## Abstract

**Objective:**

To evaluate the clinical efficacy of vestibular assessment and rehabilitation training in patients with peripheral vestibular vertigo.

**Method:**

This was a retrospective study. A total of 169 patients diagnosed with peripheral vestibular vertigo, admitted to Cangzhou People’s Hospital between January 2020 and January 2023 were divided into control group (83 cases) and observation group (86 cases). The control group received medication-based treatment, while the observation group was provided with combined treatment of medication and vestibular rehabilitation training. Assessment of recovery included the Dizziness Handicap Inventory (DHI), Vestibular Symptom Index (VSI), and Activities-specific Balance Confidence (ABC) scale before and at two, four, and eight weeks post-treatment. Psychological status, sleep quality, and life quality were evaluated. Both groups underwent the Fukuda stepping test and timed balance test.

**Result:**

At two, four, and eight weeks post-treatment, both groups exhibited significantly lower DHI-P, DHI-F, DHI-E, VSI, and ABC scores compared to pre-treatment (p<0.05). The observation group showed significantly lower DHI-P, DHI-F, DHI-E, VSI, and ABC scores than the control group at two and four weeks post-treatment (p<0.05). After treatment, both groups demonstrated reduced body deviation angles and increased time without falling in the Fukuda stepping test (p<0.05). Notably, the observation group had significantly better outcomes (p<0.05).

**Conclusion:**

In comparison to medication-based treatment alone, a combined approach involving medication treatment and vestibular rehabilitation training may demonstrate early improvements in vertigo symptoms, enhance balance capabilities, and ameliorate psychological well-being, sleep quality, and overall quality of life for patients.

## INTRODUCTION

Peripheral vestibular vertigo refers to dizziness arising from lesions in the extracranial segment of the vestibular nerve and vestibular receptors. Most patients present with auditory alterations and vestibular dysfunction, devoid of any central nervous system impairment. This condition accounts for approximately 80% to 85% of all reported vertigo cases.^1^ Presently, the precise etiology of peripheral vestibular vertigo remains elusive within clinical practice, with a multitude of factors such as trauma, infection, inflammation, viral agents, and endolymphatic hydrops being deemed as potential contributors.^2^

The mainstay of treatment for managing peripheral vestibular vertigo revolves around pharmacotherapy, wherein vestibular suppressants assume a pivotal role. Specifically, diphenhydramine hydrochloride tablets, an H1 receptor antagonist belonging to the category of vestibular suppressants, are widely used for addressing acute episodes of peripheral vestibular vertigo, effectively inducing sedation and alleviating nausea. Furthermore, this medication can effectively curb the release of histamine within the body, augment vascular permeability, and mitigate localized edema responses, thereby ameliorating the impact of the disease on the vasculature.^3^ Nevertheless, for a subset of patients grappling with peripheral vestibular vertigo, the efficacy of monotherapy employing anti-vertigo agents is circumscribed.

Additionally, due to the perpetuation of triggering factors or alternate causative agents, patients reliance solely on medication may fail to attain satisfactory vestibular compensation, resulting in recurrent bouts of vertigo, recurrent medical consultations, and a substantial detriment to daily life and occupational functioning.^4^ Consequently, the consideration of supplementary treatment modalities in tandem with pharmacotherapy assumes paramount significance. Vestibular rehabilitation training, grounded in physiotherapy centered on controlled movement, expedites the onset of vestibular compensation, thereby fostering the recuperation of impaired vestibular and balance functions.^5^ The current investigation delves into the realm of peripheral vestibular vertigo and probes the efficacy of incorporating vestibular rehabilitation training into its management.

## METHODS

This was a retrospective study. One hundred and sixty-nine patients with peripheral vestibular vertigo admitted to Cangzhou People’s Hospital from January 2020 to January 2023 were divided into control group (n=83) and observation group (n=86) according to different treatment methods. Notably, the general demographic characteristics of the control and observation groups demonstrated statistical comparability (p>0.05), as outlined in Table-I.

**Table-I:** Comparison of general demographic characteristics between the control and observation groups.

Group	Number of cases	Sex (n (%))		Average disease course (months, *χ̅*±*S*)	Average age (years,*χ̅*±*S*)

		Male	Female		
Control group	83	20(24.10)	63(75.90)	15.41±4.52	59.64±12.42
Observation group	86	26(30.23)	60(69.77)	15.86±4.61	60.32±12.73
Statistical value		0.803		0.640	0.351
*P*		0.370		0.523	0.726

### Ethical Approval

The study was approved by the Institutional Ethics Committee of Cangzhou People’s Hospital (No.: K2021-053; date: May 14, 2021), and written informed consent was obtained from all participants.

### Inclusion criteria:

- Fulfillment of the diagnostic criteria for peripheral vestibular vertigo^6^, accompanied by distinct consciousness, self-perceived self-rotation or perception of environmental rotation, sensations of dizziness or a sensation of heaviness in the head, generalized weakness, difficulties in ambulation, and in severe cases, instances of falling;
- Age ≥ 18 years;
- Voluntary provision of informed consent by the patients.

### Exclusion criteria:

- Patients with external vestibular nerve trauma accompanied by hemorrhage;
- Psychogenic dizziness, central vertigo, non-vestibular dizziness;
- Recent onset of sudden hearing loss or fluctuating auditory changes;
- Severe psychiatric disorders or malignancies;
- Individuals afflicted with severe cervical spine pathologies, fractures, cranial traumas, or other conditions precluding physical activity;
- Patients with diphenhydramine hydrochloride tablets allergy.

The control group received treatment based on medication, involving the administration of diphenhydramine hydrochloride tablets, one tablet three times a day for three consecutive days. In contrast, the observation group, in addition to the medication regimen, underwent a comprehensive vestibular rehabilitation training program. The selection of specific rehabilitation techniques was informed by an assessment of each patient’s individual vestibular function, as elaborated below:

### Enhanced Gaze Stability Training

Within this training module, patients were directed to fixate their gaze upon a business card or a small visual target positioned on the wall in front of them. While keeping their focus steadily affixed to the target, patients undertook deliberate, slow head movements in both horizontal and vertical directions. The duration of these movements was set at 0.5-1 minute for each direction. The pace of oscillations was tailored according to the patient’s prevailing condition, spanning a continuum from slow to rapid, specifically 30 times per minute-60 times per minute-90 times per minutes-120 times per minute. In instances where maintaining balance posed a challenge, patients were granted the option to widen their stance or rely on a wall for support until they could autonomously stand and execute head oscillations without any discomfort. This exercise routine was performed three times daily.

### Enhanced Postural Stability Training

This facet of the training regimen encompassed a series of exercises, including eyes-open feet-together standing, eyes-closed feet-together standing, eyes-open half-tandem stance, eyes-closed half-tandem stance, eyes-open tandem stance, eyes-closed tandem stance, eyes-open single-leg standing, and eyes-closed single-leg standing. Each exercise iteration was undertaken three times a day, with each session spanning a duration of 0.5 to one minute.

### Outcome measures:

The evaluation of recovery progress for both groups was conducted through a battery of assessment scales administered before treatment and at the intervals of two, four, and eight weeks post-treatment, including the Dizziness Handicap Inventory (DHI)^7^, the Vestibular Symptom Index (VSI)^8^, and the Activity-specific Balance Confidence (ABC) scale.^9^ The DHI scale comprised three distinct subscales: Physical (DHI-P), Functional (DHI-F), and Emotional (DHI-E), each with score ranges of 28, 36, and 32, respectively. Elevated scores were indicative of more pronounced dizziness or balance-related deficits. The VSI gauged an array of symptoms, including balance, visual sensitivity, dizziness, nausea, headache, and vertigo, assigning scores on a spectrum from 0 to 10; higher scores correlated with heightened symptom severity. In case of the ABC scale, it comprised a set of 16 items, each assessed on a scale of 0 to 100. Scores falling below 80 were suggestive of abnormal activity-related balance

### Psychological and Sleep Assessment

The psychological well-being, sleep quality, and overall life quality of participants were assessed both before and after treatment.

This comprehensive assessment encompassed the utilization of the Self-Rating Anxiety Scale (SAS)^10^, the Self-Rating Depression Scale (SDS)^11^, the Pittsburgh Sleep Quality Index (PSQI)^12^, and the 12-Item Short Form Health Survey (SF-12).^13^ The SAS and SDS yielded scores within a range of 0 to 100, where elevated scores indicated heightened severity of anxiety and depression symptoms. Within the PSQI, a total score was derived from the summation of scores across seven items, each graded from 0 to 3; a higher PSQI score correlated with increased insomnia severity. The SF-12 encompassed an array of 12 items gauging both physical and mental health aspects, yielding higher scores in alignment with enhanced life quality.

### Performance Tests

A pre-treatment and post-treatment assessment of both groups entailed the execution of the Fukuda stepping test. Closed-eye stepping balance tests were performed in a spacious, level, and tranquil setting, with deviations in body position documented during the stepping maneuvers. Simultaneously, timed balance tests were administered, capturing the total duration of maintaining equilibrium without falling across four distinct positions: eyes-open and eyes-closed heel-to-toe standing, as well as eyes-open and eyes-closed single-leg standing.

### Statistical Analysis

The software SPSS 26.0 was used for data processing, with a designated significance threshold of α= 0.05. Independent sample t test is used in this study. Measurement data were presented as mean ± standard deviation(*χ̅*±*S*) and examined using the t-test. Enumeration data were scrutinized utilizing the chi-square test. To analyze the repeated count data, repeated measures analysis of variance (ANOVA) was employed.

## RESULTS

No statistically significant differences were observed in DHI-P, DHI-F, and DHI-E scores between the two groups before treatment and at the 8-week follow-up(p>0.05). However, at two, four, and eight weeks post-treatment, both groups exhibited a noteworthy reduction in DHI-P, DHI-F, and DHI-E scores compared to their respective baseline values(p<0.05). Moreover, at two, four, and eight weeks post-treatment, the observation group displayed significantly lower scores for DHI-P, DHI-F, and DHI-E when compared to the control group(p<0.05). Notably, there were significant interaction effects observed between groups, time points, and group-time interactions in DHI-P, DHI-F, and DHI-E scores (p<0.05). Table-II.

**Table-II:** Comparison of DHI scores between the two groups (*χ̅*±*S*).

Group	Number of cases	DHI-P					

		Pre-treatment	At 2 weeks post-treatment		At 4 weeks post-treatment		At 8 weeks post-treatment
Control group	83	18.67±4.36	15.14±3.41^a^		10.48±2.61^a^		6.08±2.86^a^
Observation group	86	18.75±4.42	13.87±3.85^ab^		7.72±2.11^ab^		5.86±2.93^a^
Between-group		F = 68.693 P < 0.001					
Between-time point		F = 263.872 P < 0.001					
Group-time point		F = 14.315 P < 0.001					
Control group	83	21.35±5.45	19.02±5.02^a^	15.42±4.51^a^			6.09±2.42^a^
Observation group	86	21.42±5.62	17.25±4.86^ab^	.51±2.28^ab^			5.96±2.39^a^
Between-group		F = 89.367 P < 0.0018					
Between-time point		F = 308.574 P < 0.001					
Group-time point		F = 20.325 P < 0.001					
Control group	83	14.35±3.35	12.01±2.85^a^			5.48±2.42^a^	2.01±0.87^a^
Observation group	86	14.41±3.29	10.08±2.64^ab^			3.02±2.15^ab^	1.89±0.78^a^
Between-group		F = 65.743 P < 0.001					
Between-time point		F = 276.452 P < 0.001					
Group-time point		F = 13.756 P < 0.001					

***Note:***

a P < 0.05 compared to the pre-treatment levels; ^b^P < 0.05 compared to the post-treatment levels in the control group at the same time point.

Before and at eight weeks of treatment, no statistically significant differences were observed in VSI scores between the two groups (p>0.05). Nevertheless, a statistically significant reduction in VSI scores was evident at two, four, and eight weeks post-treatment for both groups in comparison to their baseline scores (p<0.05). Additionally, at two and four weeks post-treatment, the observation group manifested significantly lower VSI scores than the control group (p<0.05). Significant interaction effects were identified between groups, time points, and group-time interactions in VSI scores (p<0.05). Table-III.

**Table-III:** Comparison of VSI scores between the two groups (*χ̅*±*S*).

Group	Number of cases	VSI			

		Pre-treatment	At 2 weeks post-treatment	At 4 weeks post-treatment	At 8 weeks post-treatment
Control group	83	8.26±1.35	4.58±1.08^a^	2.41±0.89^a^	0.15±0.08^a^
Observation group	86	8.35±1.21	2.85±1.01^ab^	1.02±0.68^ab^	0.13±0.07^a^
Between-group		F = 70.214 P < 0.001			
Between-time point		F = 298.754 P < 0.001			
Group-time point		F = 16.158 P < 0.001			

***Note:***

a P < 0.05 compared to the pre-treatment levels; ^b^P < 0.05 compared to the post-treatment levels in the control group at the same time point.

Comparable ABC scores were documented between the two groups before treatment and at eight weeks of treatment (p>0.05). Nevertheless, the two, four, and eight weeks post-treatment witnessed a significant decrease in ABC scores for both groups when compared to their baseline scores (p<0.05). Furthermore, at two and four weeks post-treatment, the observation group exhibited significantly lower ABC scores compared to the control group (p<0.05). Notably, there were significant interaction effects noted between groups, time points, and group-time interactions in ABC scores (p<0.05). Table-IV.

**Table-IV:** Comparison of ABC scores between the two groups (*χ̅*±*S*).

Group	Number of cases	ABC			

		Pre-treatment	At 2 weeks post-treatment	At 4 weeks post-treatment	At 8 weeks post-treatment
Control group	83	68.45±8.34	71.89±6.85^a^	77.84±7.35^a^	90.08±6.45^a^
Observation group	86	67.25±8.48	76.58±7.58^ab^	85.46±6.87^ab^	90.68±7.32^a^
Between-group		F = 92.452 P < 0.001			
Between-time point		F = 315.286 P < 0.001			
Group-time point		F = 21.357 P < 0.001			

***Note:***

a P < 0.05 compared to the pre-treatment levels; ^b^P < 0.05 compared to the post-treatment levels in the control group at the same time point.

Before treatment, there were no statistically significant differences in Fukuda stepping body deviation and balance time between the two groups (p>0.05). However, post-treatment assessments revealed a noteworthy reduction in Fukuda stepping body deviation and an increase in balance time for both groups in contrast to their baseline measurements (p<0.05). Furthermore, the observation group showed diminished Fukuda stepping body deviation and prolonged balance time when compared to the control group (p<0.05). Table-V.

**Table-V:** Comparison of Fukuda stepping and timed balance test results between the two groups (*χ̅*±*S*).

Group	Number of cases	Fukuda stepping test (degree,°)		Timed balance test (minutes)	

		Pre-treatment	Post-treatment	Pre-treatment	Post-treatment
Control group	83	87.45±8.54	38.82±3.75^a^	28.65±2.95	120.45±6.71^a^
Observation group	86	88.51±7.68	28.47±3.05^a^	28.85±3.02	130.25±7.25^a^
t		0.013	19.716	0.435	9.111
P		0.990	<0.001	0.664	<0.001

***Note:***

a P < 0.05 when comparing the pre-and post-treatment test results within each group.

Before treatment, the two groups did not differ greatly in SAS, SDS, PSQI, and SF-12 scores (p>0.05). After treatment, both groups demonstrated a significant decrease in SAS, SDS, and PSQI scores, coupled with an increase in SF-12 scores compared to their respective baseline values (p<0.05). Furthermore, the observation group displayed significantly lower SAS, SDS, and PSQI scores, along with higher SF-12 scores, in comparison to the control group (p<0.05). Table-VI.

**Table-VI:** Comparison of psychological status, sleep quality, and life quality between the two groups (*χ̅*±*S*).

Group	Number of cases	SAS		SDS	

		Pre-treatment	Post-treatment	Pre-treatment	Post-treatment
Control group	83	58.78±6.75	42.23±5.28^a^	56.23±5.59	43.58±5.21^a^
Observation group	86	58.85±7.02	35.47±5.12^a^	56.42±5.47	34.15±4.35^a^
*t*		0.066	8.450	0.223	12.790
*P*		0.947	<0.001	0.824	<0.001

***Group***	***Number of cases***	***PSQI***		***SF-12***	

		***Pre-treatment***	***Post-treatment***	***Pre-treatment***	***Post-treatment***

Control group	83	16.21±1.25	7.58±1.42^a^	48.36±7.25	82.54±5.36^a^
Observation group	86	16.32±1.18	4.01±1.36^a^	48.48±7.14	93.58±5.14^a^
*t*		0.588	16.694	0.108	13.669
*P*		0.557	<0.001	0.914	<0.001

***Note:***

a P < 0.05 when comparing the pre- and post-treatment test results within each group.

## DISCUSSION

In this study, the drug intervention employed diphenhydramine hydrochloride, a compound that has demonstrated efficacy in enhancing vascular permeability, facilitating inner ear circulation, mitigating vascular spasm of the inner ear and vertebrobasilar arteries, and even alleviating or eradicating endolymphatic hydrops, thereby relieving symptoms of dizziness. At the core of vestibular rehabilitation training resides the fundamental principle of harnessing the brain’s plasticity, adaptability, and compensatory mechanisms through the manipulation of the vestibulospinal and vestibulo-ocular reflexes.

By integrating activities like balance and gait training, gaze stability exercises, walking regimens, and habituation training, this approach fosters vestibular compensation, ultimately assuaging dizziness symptoms and reinstating equilibrium.^14^ The DHI and VSI scales represent widely employed tools for evaluating the severity of clinical symptoms and gauging treatment outcomes in patients suffering from dizziness. These instruments exhibit strong reliability in assessing peripheral vestibular vertigo.^14^ Concurrently, the ABC scale serves as a means to appraise a patient’s confidence in maintaining stability during a spectrum of tasks, and its reliability has been substantiated for use in patients afflicted with peripheral vestibular vertigo.^15^

Throughout this study, the DHI-P, DHI-F, DHI-E, VSI, and ABC scores for both groups consistently exhibited reduction after treatment at the two, four, and eight weeks post-treatment in comparison to baseline. Particularly noteworthy, the observation group displayed even lower scores when contrasted with the control group at weeks two and four post-treatment. This observation is consistent with the conclusions drawn from a study conducted by Lin et al.^16^, which lends support to the proposition that a combination of vestibular rehabilitation training and medication can effectively manage dizziness symptoms, bolster balance capacities, and enhance confidence in maintaining equilibrium.

Furthermore, the outcomes of this investigation unveiled a reduction in body deviation angles during the Fukuda stepping test and an elongated duration of maintaining balance without falling for both groups following the treatment regimen. Notably, the observation group exhibited notably superior outcomes when compared with the control group. These findings serve to fortify the proposition that the combination of vestibular rehabilitation training with medication bestows a pronounced enhancement in the balance status of individuals with peripheral vestibular vertigo.

In recent years, as the depth of exploration into peripheral vestibular vertigo has expanded, a growing recognition has emerged regarding the frequently encountered sleep disturbances and negative emotional experiences endured by patients with this condition. A study conducted by Cengiz DU et al.^17^ brought to light significant disparities in scores for the PSQI and the Hospital Anxiety and Depression Scale between individuals with dizziness and their healthy counterparts, suggesting that patients with dizziness are often burdened with adverse emotions such as anxiety and depression, in tandem with experiencing sleep disturbances. Additionally, research undertaken by Molnár A et al.^18^ revealed that approximately 42.3% of individuals with dizziness displayed symptoms indicative of depression, a manifestation that exerted a notable impact on their overall quality of life. Likewise, the investigation conducted by Mutlu B et al.^19^ underscored elevated scores on the Beck Depression Inventory and PSQI among patients with peripheral vestibular vertigo in comparison to a healthy control group, indicating that these individuals frequently experience varying degrees of psychological stress and a diminished quality of sleep.

Currently, uncertainty prevails regarding the potential of vestibular rehabilitation training to ameliorate the psychological and sleep status of individuals with vestibular vertigo. This study was designed to bridge this gap in understanding, and the findings unveiled a noteworthy pattern: after treatment, both study groups showed diminished scores on the scales SAS, SDS, and PSQI, while concurrently witnessing elevated scores on the SF-12 in comparison to baseline measurements. Remarkably, the observation group emerged with lower SAS, SDS, and PSQI scores, coupled with higher SF-12 scores in contrast to the control group.

These findings underscore the potential of integrating vestibular rehabilitation training with medication to not only alleviate dizziness symptoms but also engender a notable enhancement in psychological well-being, sleep quality, and overall life quality. This observed amelioration can be ascribed to a multitude of factors. Vestibular rehabilitation training exerts effective control over early-stage dizziness symptoms, augments patients’ mastery over balance control, and instills a heightened sense of confidence in maintaining equilibrium. This cumulative impact serves as a catalyst for improved psychological well-being, augmented sleep quality, and more favorable overall quality of life.

Moreover, the meaningful interaction between healthcare practitioners and patients during the course of vestibular rehabilitation fosters a deeper comprehension of the ailment, gradually dispelling apprehensions of dire consequences and significantly bolstering patients’ self-assurance in managing their condition. This positive shift in mindset creates an enabling environment for the restoration of psychological equilibrium and sleep quality, thus culminating in an overarching enhancement in the quality of life.

### Limitations of the study

However, the conclusions are still controversial due to the small sample size and different criteria used by the current studies. As such in view of the research design of single-center study with smaller sample size, multi-center studies are needed based on larger sample size for further verification.

## CONCLUSION

To summarize, compared to the sole use of medication, the combination therapy of drug intervention and vestibular rehabilitation training may present an avenue for early alleviation of dizziness symptoms, an augmentation of equilibrium capabilities, and a palpable positive impact on psychological well-being, sleep quality, and overall life quality.

### Authors’ Contributions:

**HW** and **JZ:** Carried out the studies, participated in collecting data, drafted the manuscript, are responsible and accountable for the accuracy and integrity of the work.

**FL:** Participated in acquisition, analysis, or interpretation of data and drafting the manuscript.

**CF** and **NZ:** Performed the statistical analysis and participated in its design.

All authors read and approved the final manuscript.
